# Tightly focused optical skyrmions and merons formed by electric-field vectors with prescribed characteristics

**DOI:** 10.1515/nanoph-2023-0741

**Published:** 2024-01-03

**Authors:** Yongxi Zeng, Yanzhong Yu, Xi Shen, Jian Chen, Qiwen Zhan

**Affiliations:** School of Optical-Electrical and Computer Engineering, University of Shanghai for Science and Technology, Shanghai 200093, China; College of Physics and Information Engineering, Quanzhou Normal University, Fujian 362000, China; Fujian Provincial Key Laboratory for Advanced Micro-Nano Photonics Technology and Devices & Key Laboratory of Information Functional Material for Fujian Higher Education, Quanzhou 362000, China; Shanghai Key Laboratory of Modern Optical System, University of Shanghai for Science and Technology, Shanghai 200093, China

**Keywords:** optical skyrmion, optical meron, topology, dipole radiation, 4*π*-focusing system

## Abstract

Optical skyrmions, which are topological quasi-particles with nontrivial electromagnetic textures, have garnered escalating research interest recently for their potential in diverse applications. In this paper, we present a method for generating tightly focused optical skyrmion and meron topologies formed by electric-field vectors under 4*π*-focusing system, where both the topology types (including Néel-, Bloch-, intermediate- and anti-skyrmion/meron) and the normal direction of the two-dimensional topology projection plane can be tailored at will. By utilizing time-reversal techniques, we analytically derive the radiation pattern of a multiple concentric-ring array of dipoles (MCAD) to obtain the required illumination fields on the pupil planes of the two high numerical aperture lenses. The Deby vector diffraction integral theory is employed to calculate the corresponding tightly focused field, and their topology characteristics are quantitatively evaluated by the electric-field vector distribution. The results demonstrate that arbitrary electric-field based skyrmion and meron can be conveniently generated by adjusting the oscillation direction of each dipole in the MCAD and the normal direction of the dipole array. The generated optical topologies with fully controllable degrees of freedom provide potential applications in optical information processing, transmission, and storage.

## 1 Introduction

In 1961, British physicist Skyrme proposed a quasi-particles structure with topological protection while investigating the unified field theory of interactions between mesons and baryons [1], [2]. Later, the emerging quasi-particle was mathematically termed as “skyrmion” [3]. Subsequent studies have demonstrated the existence of skyrmions in various environments, including quantum fields [4], solid-state physics [5], [6], liquid-crystal materials [7], and Bose–Einstein condensates [8]. Particularly in condensed matter physics, magnetic skyrmions have received extensive research attention due to their potential applications in high-capacity information encoding and low-energy magnetic storage [9], [10], [11], [12].

It was not until 2018 that one photonic analogies of magnetic skyrmion, defined in terms of the electric-field vector variation, was reported firstly in Science [13]. It was formed by interfering multiple surface plasmon polariton (SPP) waves, observed by imaging SPP using phase-resolved near-field optical microscopy, thereby opening up a frontier research topic in the optical domain. Over the past five years, scholars have reported a variety of optical skyrmions constructed by different object vectors, such as spin angular momentum of confined free-space waves [14], [15], [16], stokes vectors of paraxial beams [17], [18], and magnetic-field vectors of pulse-propagating light [19]. These constructed optical skyrmions hold tremendous potential in various applications, including subwavelength microscopy [14], nanoscale metrology [14], [20], topological Hall devices [15] and ultrafast vector imaging [21], significantly expand the frontiers of modern fundamental and applied physics research [22], [23]. Among these different applications, the most crucial aspect is the achievement of interactions between light and matter at the micro- and nano-scale, which enable the exploration of novel physical phenomena.

To the best of our knowledge, a limited body of literature dedicated to the construction of electric-field vector skyrmion and meron topologies in tightly focused fields with arbitrary prescribed characteristics, including simultaneous control of both the optical topologies and their normal directions of two-dimensional topology projection planes. We present a methodology generating a variety of electric-field skyrmion and meron topologies with arbitrarily specified characteristics in this paper, thereby enhancing their controllable degrees of freedom. The construction of these optical topologies is accomplished by combining time-reversal techniques, array antenna radiation theory, and Debye vector diffraction integral theory. The proposed methods are expounded upon in Section 2, followed by the presentation of numerical simulations and ensuing discussions in Section 3. A succinct summary is subsequently furnished in Section 4.

## 2 Methods

**Figure 1:**
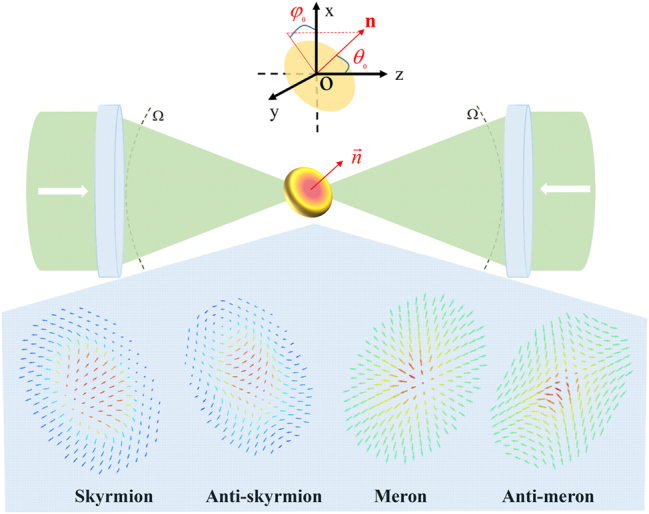
Schematic illustration of optical setup to generate electric-field skyrmions and merons in the focal region.

Figure 1 depicts the optical setup utilized in our method, termed as the 4*π*-focusing system [24], [25]. It consists of two identical high numerical aperture (NA) objective lenses, with their focal points overlapping and their optical axes aligned. The focal point of the objective lens is selected as the origin of the global Cartesian coordinates, with its *z*-axis coinciding with the optical axis. This 4*π*-focusing system is utilized to converge the incident fields from both sides of the entrance pupil planes, resulting in the formation of the desired focused field in the confocal region.

Here we utilize a multiple concentric-ring array of dipoles (MCAD) to mimic the radiation source for calculating the required pupil plane field, carefully arrange the orientation of each dipole to simulate the electric-field vector distribution in the focal field. The multiple concentric-ring array structure is employed to achieve approximately equal spacing between dipoles on the same ring and equal distance from the origin for each dipole on the same ring, accurately mimicking the electric-field polarization structure of the skyrmionic topology. The MCAD is arranged in the prescribed plane passing through the origin, as indicated by the yellow shaded area in Figure 1. The normal direction of this plane is denoted as **n**, with its direction defined by $\left(\theta_{0},\varphi_{0}\right)$ , where *θ*_0_ represents the polar angle between **n** and the *z*-axis, and *φ*_0_ represents the azimuth angle between its projection onto the *x*–*y* plane and the *x*-axis.

**Figure 2:**
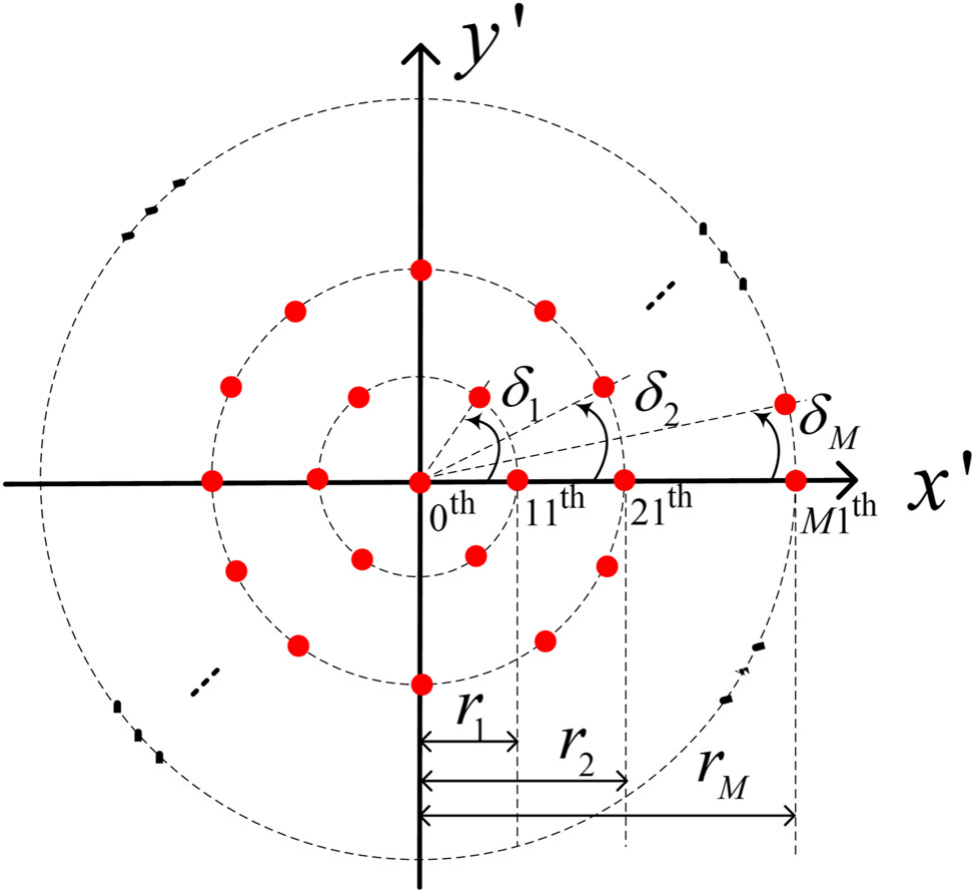
Arrangement of MCAD.

Now, we establish a local Cartesian coordinates, which is formed by rotating the *z*-axis of the global Cartesian coordinates around the origin to the normal direction **n**. The normal direction **n** serves as the *z*′-axis of the local Cartesian coordinates, while the *x*- and *y*-axes of the global Cartesian coordinates are rotated synchronously to align with the new *x*′- and *y*′-axes, respectively. Subsequently, we carefully arrange a MCAD in the *x*′–*y*′ plane, as depicted in Figure 2.

In Figure 2, the red solid dots represent the dipoles. One dipole labeled as 0th dipole is arranged at the origin with its oscillation direction align along the *z*′-axis, which corresponds to the normal vector **n** in Figure 1. The numbering convention for the other dipoles is denoted as *mn*th, where *m* indicates the ring number starting from the origin and *n* represents the index of a dipole in *m*th ring, counting counterclockwise from the dipole located on the *x*′-axis. M refers to the labeling of the outermost ring. *δ*_1_, *δ*_2_, …, *δ*_*M*_ denote the angular spacing of neighboring dipoles on each rings. *r*_1_, *r*_2_, …, *r*_*M*_ represent the radii of the 1th, 2th, …, *m*th rings, respectively. It is important to note that dipoles on the same ring share identical oscillation polar angles, while their oscillation azimuth angles are contingent upon the dipole’s position and the desired topologies of electric-field vectors. The number of dipoles from the 1th to the *M*th ring are denoted as *N*_1_, *N*_2_, …, *N*_*M*_, where *N*_1_ × *δ*_1_ = *N*_2_ × *δ*_2_ = … = 2*π*.

The total radiation field of the MCAD results from the coherent superposition of individual dipole radiation fields located at distinct positions with varying oscillation directions. Additionally, each dipole’s radiation pattern can be further decomposed into the coherent combination of dipole radiation fields aligned with the three principal axes of global Cartesian coordinates. According to electromagnetic radiation theory [26], [27], the expressions for calculating the radiation fields $E_{dx}\left(\theta ,\varphi \right)$ , $E_{dy}\left(\theta ,\varphi \right)$ and $E_{dz}\left(\theta ,\varphi \right)$ emanating from the three dipoles positioned along the *x*-, *y*-, and *z*-axes of the global Cartesian coordinates on the spherical surface Ω subsequent to the objective lenses can be written as follows [28]

(1) $$\left[\begin{array}{c} E_{dx}\left(\theta ,\varphi \right)\\ E_{dy}\left(\theta ,\varphi \right)\\ E_{dz}\left(\theta ,\varphi \right) \end{array}\right]=C\left[\begin{array}{c} \cos\theta \cos\varphi e_{\theta }-\sin\varphi e_{\varphi }\\ \cos\theta \sin\varphi e_{\theta }+\cos\varphi e_{\varphi }\\ -\sin\theta e_{\theta } \end{array}\right],$$

where *C* = *jωμ*_0_ *I*_0_ *l*e^−*jkf*^/4*πf* represents a factor that is independent of the directional properties of the radiation field, *f* denotes the focal length of the lens, *θ* is defined in relation to the global Cartesian coordinates as the focusing angle measured from the *z*-axis and *φ* is the azimuthal angle. The directional angles of the *x*′-, *y*′- and *z*′-axes in global Cartesian coordinates are denoted as $\left(\alpha_{1},\beta_{1},\gamma_{1}\right)$ , $\left(\alpha_{2},\beta_{2},\gamma_{2}\right)$ and $\left(\alpha_{3},\beta_{3},\gamma_{3}\right)$ , respectively, where subscripts 1, 2 and 3 correspond to the angles with respect to the *x*-, *y*- and *z*-axes, respectively. Consequently, the radiation fields emanating from the dipoles situated along the *x*′-, *y*′-, and *z*′-axes of local Cartesian coordinates can be expressed by the direction cosines matrix, as follows:

(2) $$\begin{array}{ll} \left[\begin{array}{c} E_{dx^{\prime }}\left(\theta ,\varphi \right)\\ E_{dy^{\prime }}\left(\theta ,\varphi \right)\\ E_{dz^{\prime }}\left(\theta ,\varphi \right) \end{array}\right] & =\left[\begin{array}{ccc} \cos\alpha_{1} & \cos\beta_{1} & \cos\gamma_{1}\\ \cos\alpha_{2} & \cos\beta_{2} & \cos\gamma_{2}\\ \cos\alpha_{3} & \cos\beta_{3} & \cos\gamma_{3} \end{array}\right]\left[\begin{array}{c} E_{dx}\left(\theta ,\varphi \right)\\ E_{dy}\left(\theta ,\varphi \right)\\ E_{dz}\left(\theta ,\varphi \right) \end{array}\right]\\ & =\left[\begin{array}{ccc} \cos\theta_{0}\cos^{2}\varphi_{0}+\sin^{2}\varphi_{0} & \left(\cos\theta_{0}-1\right)\sin\varphi_{0}\cos\varphi_{0} & -\sin\theta_{0}\cos\varphi_{0}\\ \left(\cos\theta_{0}-1\right)\sin\varphi_{0}\cos\varphi_{0} & \cos\theta_{0}\sin^{2}\varphi_{0}+\cos^{2}\varphi_{0} & -\sin\theta_{0}\sin\varphi_{0}\\ \sin\theta_{0}\cos\varphi_{0} & \sin\theta_{0}\sin\varphi_{0} & \cos\theta_{0} \end{array}\right]\left[\begin{array}{c} E_{dx}\left(\theta ,\varphi \right)\\ E_{dy}\left(\theta ,\varphi \right)\\ E_{dz}\left(\theta ,\varphi \right) \end{array}\right]. \end{array}$$

The radiation field of each dipole situated in the *x*′–*y*′ plane of Figure 2 can be derived through the coherent combination of the radiation fields of the three dipoles, as expressed by Equation (2). The total radiation field of the MCAD is obtained by coherently summing of the radiation fields of each dipole, as follows:

(3) $$E_{\text{all}}\left(\theta ,\varphi \right)=E_{0}+\overset{M}{\underset{m=1}{\sum }}\overset{N_{m}}{\underset{n=1}{\sum }}AF_{mn}E_{mn},$$

where

(4) $$E_{0}=E_{dz^{\prime }}\left(\theta ,\varphi \right),$$

(5) $$AF_{mn}=\exp\left\{-jkr_{m}\left[\begin{array}{c} \left(\cos\alpha_{1}\cos\varphi_{p\text{\_}mn}+\cos\alpha_{2}\sin\varphi_{p\text{\_}mn}\right)\sin\theta \cos\varphi +\\ \left(\cos\beta_{1}\cos\varphi_{p\text{\_}mn}+\cos\beta_{2}\sin\varphi_{p\text{\_}mn}\right)\sin\theta \sin\varphi +\\ \left(\cos\gamma_{1}\cos\varphi_{p\text{\_}mn}+\cos\gamma_{2}\sin\varphi_{p\text{\_}mn}\right)\cos\theta \end{array}\right]\right\},$$

(6) $$\begin{array}{ll} E_{mn} & =\sin\theta_{d\text{\_}m}\cos\varphi_{d\text{\_}mn}E_{dx^{\prime }}\left(\theta ,\varphi \right)+\sin\theta_{d\text{\_}m}\sin\varphi_{d\text{\_}mn}E_{dy^{\prime }}\left(\theta ,\varphi \right)\\ & \;+\cos\theta_{d\text{\_}m}E_{dz^{\prime }}\left(\theta ,\varphi \right). \end{array}$$

*E*_0_ refers to the radiation field of 0th dipole, with oscillation direction $\left(\theta_{0},\varphi_{0}\right)$ , that is, $E_{dz^{\prime }}\left(\theta ,\varphi \right)$ . $\left(AF_{mn}E_{mn}\right)$ represent the radiation field of the *mn*th dipole, with its oscillation direction along $\left(\theta_{d\text{\_}m},\varphi_{d\text{\_}mn}\right)$ . The radiation field consists of two components, *E*_*mn*_ represents the radiation field of the dipole when it is situated at the origin, which can be acquired by coherently summing the radiation fields of three dipoles aligned with the principal axes of the local Cartesian coordinates, as shown by Equation (6). It is important to note that $\left(\theta_{d\text{\_}m},\varphi_{d\text{\_}mn}\right)$ are defined in relation to the local Cartesian coordinates. *AF*_*mn*_ and *φ*_*p*_*mn*_ represent position factor of the *mn*th dipole in the global Cartesian coordinates and its positional azimuthal angle in the *x*′–*y*′ plane, respectively.

The skyrmion topology is determined by its polarity *p*, vorticity *v*, and helicity *φ*_*r*_ [23], [29]. Polarity and vorticity describe the orientation variations of the out-of-plane and in-plane electric-field vector, respectively, and helicity accounts for the initial phase of the in-plane electric-field vector. In our proposed method, these parameters relate to the oscillation direction and position of each dipole, as follows:

(7) $$\theta_{d\text{\_}m}=\left(p+1\right)\pi /2-p\theta_{d\text{\_}m}^{\prime },$$

(8) $$\varphi_{d\text{\_}mn}=v\cdot \varphi_{p\text{\_}mn}+\varphi_{r}.$$

One note that the oscillation polar angle *θ*_*d*_*m*_ of dipoles on the same ring is identical, while *φ*_*d*_*mn*_ of each dipole is dependent on its specific position on the ring and the vorticity *v* and helicity *φ*_*r*_ of the desired topological structure. As for the polarity *p*, if the 0th dipole of *θ*_0_ = *π*, the outermost ring is 0, then *p* = 1; vice versa, *p* = −1. $\theta_{d\text{\_}m}^{\prime }$ denotes the initial oscillation polar angle of the dipoles in the *m*th ring. When the polarity is inverted, the oscillation polar angle of the dipoles takes the complementary angle of the initial value, as expressed in Equation (7).

The total radiations field of the MCAD can be decomposed into a radial component **e**_*θ*_ and an azimuthal component **e**_*φ*_ on the spherical surface Ω after the objective lenses, as expressed by the following equation:

(9) $$E_{\text{all}}\left(\theta ,\varphi \right)=C\left[R_{\theta }\left(\theta ,\varphi \right)e_{\theta }+R_{\varphi }\left(\theta ,\varphi \right)e_{\varphi }\right],$$

where $R_{\theta }\left(\theta ,\varphi \right)$ and $R_{\varphi }\left(\theta ,\varphi \right)$ represent the expressions for the radial and azimuthal components, which can be derived from Equation (3). By employing two typical high NA lenses that satisfies the sine condition, $E_{\text{all}}\left(\theta ,\varphi \right)$ is collected and collimated. Considering the bending effect of lenses on light rays, the incident field at the entrance pupil can be expressed with polar coordinates $\left(\rho ,\varphi \right)$ as follows [30], [31]

(10) $$E_{\text{in}}\left(\rho ,\varphi \right)=C\left[R_{\theta }\left(\theta ,\varphi \right)e_{\rho }+R_{\varphi }\left(\theta ,\varphi \right)e_{\varphi }\right]/\sqrt{\cos\theta },$$

where $\sqrt{\cos\theta }$ denotes the apodization function of the lenses, and *ρ* = *f* sin*θ*. Employing time-reversal technique, the incident field described in Equation (10) is reversed and phase-shifted by *π* on both sides of the pupil planes for inverse focusing. Here, we utilize Deby vector diffraction integral theory [32], [33] to quantitatively evaluate the focal field in the confocal region,

(11) $$\begin{array}{ll} E_{\text{foc}}\left(r,\phi ,z\right) & =\frac{j}{\lambda }\int_{0}^{\theta_{\max}}\int_{0}^{2\pi }E_{\;\Omega }\left(\theta ,\varphi \right)\\ & \;\times e^{\left[-jkr\sin\theta \cos\left(\varphi -\phi \right)-jkz\cos\theta \right]}\sin\theta d\theta d\varphi \\ & \;+\frac{j}{\lambda }\int_{\pi }^{\pi +\theta_{\max}}\int_{0}^{2\pi }E_{\;\Omega }\left(\theta ,\varphi \right)e^{j\pi }\\ & \;\times e^{\left[-jkr\sin\theta \cos\left(\varphi -\phi \right)-jkz\cos\theta \right]}\sin\theta d\theta d\varphi , \end{array}$$

where (*r*, *ϕ*, *z*) is the cylindrical coordinate of the focal field, and $r=\sqrt{x^{2}+y^{2}}$ , *ϕ* = cos^−1^(*x*/*r*). The two integrals on the right side of Equation (11) are used to calculate the focusing fields of the left and right lenses, respectively. $E_{\;\Omega }\left(\theta ,\varphi \right)$ is the refracted field corresponding to $E_{\text{in}}\left(\rho ,\varphi \right)$ , which can be derived as

(12) $$E_{\Omega }\left(\theta ,\varphi \right)=C\left\{\begin{array}{c} \;\;\;\left[R_{\theta }\left(\theta ,\varphi \right)\cos\theta \cos\varphi -R_{\varphi }\left(\theta ,\varphi \right)\sin\varphi \right]e_{x}\\ +\left[R_{\theta }\left(\theta ,\varphi \right)\cos\theta \sin\varphi +R_{\varphi }\left(\theta ,\varphi \right)\cos\varphi \right]e_{y}\\ +R_{\theta }\left(\theta ,\varphi \right)\sin\theta e_{z} \end{array}\right\}.$$

To characterize the topological properties of the tightly focused electric field, we calculate its skyrmion number, which represents the number of times the electric-field vector wraps around a unit sphere, expressed as [13], [34]

(13) $$S_{N}=\frac{1}{4\pi }\underset{A}{\iint }E_{\text{foc\_u}}\cdot \left(\frac{\partial E_{\text{foc\_u}}}{\partial x}\times \frac{\partial E_{\text{foc\_u}}}{\partial y}\right)dxdy,$$

where *E*_foc_u_ represents unit electric-field vector of the focused field $E_{\text{foc}}\left(r,\phi ,z\right)$ , and *A* denotes the chosen two-dimensional integration domain. Due to the radial symmetry of the vector structure, skyrmion number represented by Equation (13) can be simplified as *S*_*N*_ = *p* ⋅ *v* [23]. Utilizing the aforementioned model, precise control over the oscillation direction of individual dipoles in MCAD can be achieved, enabling manipulation for the desired topological textures of the electric-field vectors.

## 3 Results and discussions

In the third section, we employ the method proposed in the second section to generate several optical topologies formed by electric-field vector with arbitrary prescribed characteristics. To simplify the calculations, we normalize the parameter *C*, which is independent of the dipole’s radiation pattern. We employ two objective lens that conforms to the sine condition to form a 4*π*-focusing system. The numerical aperture of the objective lenses is set to 1 in order to fully converge the radiation field of the MCAD, and it can be achieved by a parabolic mirror [35]. We utilize the optimized MCAD arrangement parameters to determine the position of each dipole: (i) a dipole is situated at the origin with oscillation direction $\left(\theta_{0},\varphi_{0}\right)$ aligned with the normal **n** to the MCAD arrangement plane; (ii) the MCAD consists of five concentric rings with radii *r*_1_ = 0.18*λ*, *r*_2_ = 0.36*λ*, *r*_3_ = 0.54*λ*, *r*_4_ = 0.72*λ*, and *r*_5_ = 0.90*λ*; (iii) the number of dipoles in each ring is *N*_1_ = 6, *N*_2_ = 12, *N*_3_ = 18, *N*_4_ = 24 and *N*_5_ = 30, that is, *δ*_1_ = *π*/3, *δ*_2_ = *π*/6, *δ*_3_ = *π*/9, *δ*_4_ = *π*/12, and *δ*_5_ = *π*/15.

### 3.1 Highly confined optical skyrmion

**Figure 3:**
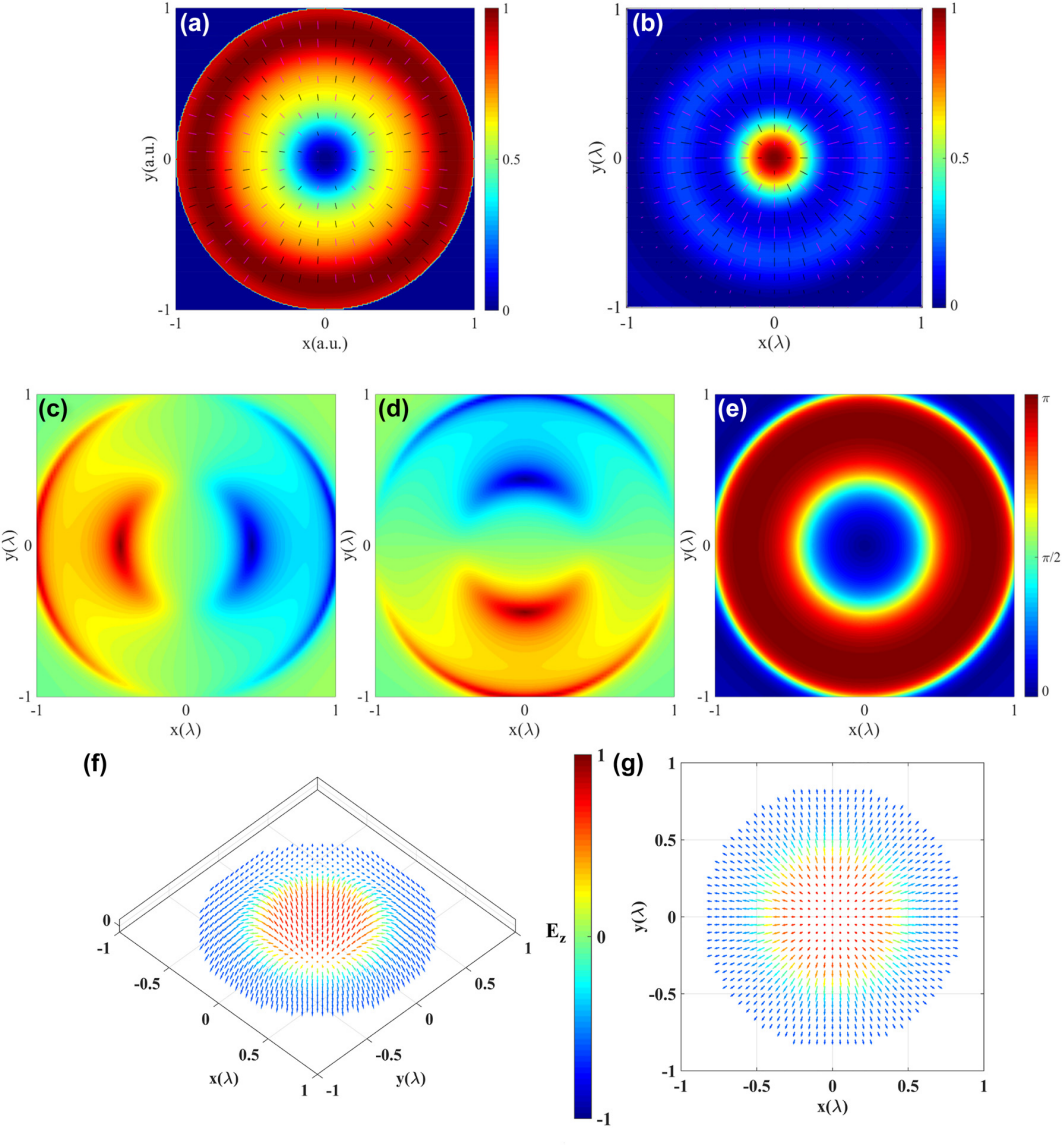
Néel-type optical skyrmion formed by electric field in *x*–*y* plane. (a) Intensity and polarization distribution of the required incident field, (b) intensity and polarization distribution in the focal plane, (c)–(e) directional angles of the electric-field vectors, (f) and (g) are the three dimensional distribution of electric-field vector and its in-plane component in focal plane, the arrows indicate the orientation of the normalized electric-field and color coded with its *z*-component.

Without loss of generality, we take the generation of a typical Néel-type electric-field vector Skyrmion located at the focal plane as the first instance. We set *p* = −1, *v* = 1, *φ*_*r*_ = 0 and $\left(\theta_{0},\varphi_{0}\right)=\left(0,0\right)$ , that is, *φ*_*d*_*mn*_ = *φ*_*p*_*mn*_, and the MCAD is arranged on *x*–*y* plane of the global Cartesian coordinates. The initial polar angles of the dipole oscillation directions in the first to fifth rings are $\theta_{d\text{\_}1}^{\prime }=\pi /5$ , $\theta_{d\text{\_}2}^{\prime }=2\pi /5$ , $\theta_{d\text{\_}3}^{\prime }=3\pi /5$ , $\theta_{d\text{\_}4}^{\prime }=4\pi /5$ and $\theta_{d\text{\_}5}^{\prime }=\pi$ . By employing Equation (10), we calculate the required incident field. Subsequently, we substitute it into Equation (11) to quantitatively evaluate the electric field within the focal region. The desired illumination and the characteristics of the electric-field vector skyrmion constructed on the *x*–*y* plane are illustrated in Figure 3.

Figure 3(a) illustrates the intensity and polarization distribution of the required incident field. It can be observed that the intensity exhibits a ring-shaped symmetric distribution, with weaker intensity in the central region and stronger intensity on the outer side. The polarization distribution shows a radial distribution pattern. The center of the incident field corresponds to a zero-intensity point and a polarization singularity. The pattern of their centrally symmetric distribution is related to the centrally symmetric arrangement of the MCAD in the *x*–*y* plane. The incident field undergoes diffraction by two high NA objective lenses, resulting in a central bright and peripheral weak ring-shaped distribution, as well as a radial polarization pattern.

Figure 3(b) illustrates the intensity and polarization distribution in the focal plane. The orientation of the electric-field vector in the focal plane can be quantitatively evaluated using their directional angles (*α*, *β*, *γ*), as shown in Figure 3(c)–(e). Here, (*α*, *β*, *γ*) represent the angles between the electric-field vector and the *x*-, *y*- and *z*-axis, respectively. It can be observed that the distribution patterns of *α* and *β* are similar and shifted by *π*/2 from each other. *α* approaches *π*/2 when *x* = 0, and *β* approaches *π*/2 when *y* = 0. These patterns arise from the arrangement of the MCAD. Particularly notable is the center point of the *γ* pattern, which is at 0. This is because the oscillation of the 0th dipole in the MCAD aligns with the *z*-axis. As the radius increases, *γ* gradually increases. When *r* = 0.42*λ*, *γ* is *π*/2, and when *r* = 0.84*λ*, *γ* is *π*. Therefore, the direction of the electric-field vector completes a full flip from “upward” at the center to “downward” at the periphery. Figure 3(f) visually demonstrates a complete flip of the electric-field vector within the region of radius 0.84*λ* in the focal plane, and the color of the arrows represents the angle between the electric-field vector and the normal vector **n**, that is the *z*-component of the unit electric-field vector. Figure 3(g) depicts the distribution of the in-plane of the electric-field vector in the focal plane. As the helicity *φ*_*r*_ is set to 0, the distribution exhibits an outward radial pattern. The spatial distribution of the electric-field vector exhibits a pattern similar to the Néel-type magnetic skyrmions observed in magnetic materials. To further characterize the distribution characteristics of the electric-field vector, we utilize the Equation (13) to calculate its skyrmion number is −0.9386 (when radius is 0.84*λ*), which confirms the successful construction of an effective Néel-type electric-field vector skyrmion in the focal plane.

### 3.2 Tunable optical skyrmion and meron topologies

**Figure 4: j_nanoph-2023-0741_fig_004:**
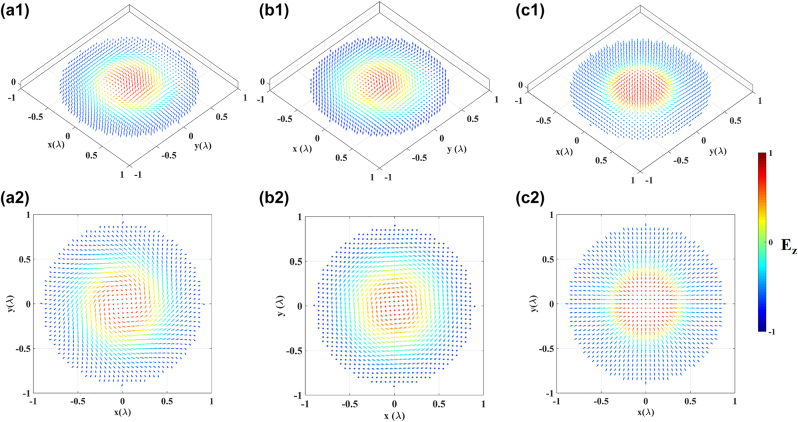
Skyrmions with prescribed helicity (color coded for the value of its *z*-component). (a1) *φ*_*r*_ = *π*/4, (b1) *φ*_*r*_ = *π*/2, (c1) *φ*_*r*_ = *π*, (a2)–(c2) are the in-plane component distribution of (a1), (b1) and (c1).

In the second instance, we generate different topologies of electric-field vectors on the *x*–*y* plane. Firstly, based on the parameters from the first instance, we can achieve a continuous deform of the electric-field vector skyrmion from the Néel-type to Bloch-type texture by adjusting the helicity *φ*_*r*_. When *φ*_*r*_ is equal to 0 or *π*, it corresponds to the Néel-type texture, and when *π*/2 or 3*π*/2, it corresponds to the Bloch-type texture [34]. Other values of *φ*_*r*_ represents intermediate-skyrmions. Figure 4 illustrates three cases chosen when *φ*_*r*_ takes on the values *π*/4, *π*/2 and *π*, depicting the three-dimensional distribution of the electric-field vector and its in-plane component distribution in the *x*–*y* plane. From Figure 4(a2)–(c2), it is evident that the in-plane component of the electric-field vector exhibits an initial phase shift determined by *φ*_*r*_, while the out-of-plane component remains unchanged and is determined by the dipoles’ oscillation polar angle. This deformation of the topologies aligns with the theoretical expectations. Furthermore, the respective skyrmion numbers are obtained by Equation (13) to be −0.9510, −0.9881, and −0.9386, which indicates the effectiveness of these topologies as skyrmionic structures. The nearly unchanged skyrmion numbers also demonstrate the topological stability of the skyrmions.

**Figure 5: j_nanoph-2023-0741_fig_005:**
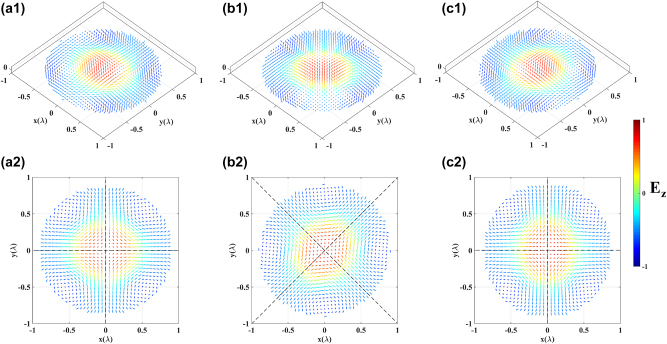
Anti-skyrmions with prescribed helicity. (a1) *φ*_*r*_ = 0, (b1) *φ*_*r*_ = *π*/2, (c1) *φ*_*r*_ = *π*, (a2)–(c2) are the top view of (a1), (b1) and (c1), respectively.

Secondly, still based on the parameters of the first instance, we adjusted the vorticity *ν* to −1, resulting in the formation of anti-skyrmions, where their helicity *φ*_*r*_ can also be arbitrarily prescribed. Figure 5 illustrates the three-dimensional topological structures and the in-plane component distributions for three cases when *φ*_*r*_ takes on the values 0, *π*/2 and *π*. The respective skyrmion numbers are 1.0317, 1.0143, and 1.0317, which also demonstrate their topological stability. It is worth noting that the helicity has a different meaning in this case, which differs from that of *v* = 1 skyrmions. It does not represent a global offset of the in-plane component of the electric-field vector, but rather distinguishes between two axes, along which the anti-skyrmion exhibits Néel-type skyrmionic profiles with helicity of 0 and *π*, respectively [29], as depicted by the black dashed lines in Figure 5(a2)–(c2).

**Figure 6:**
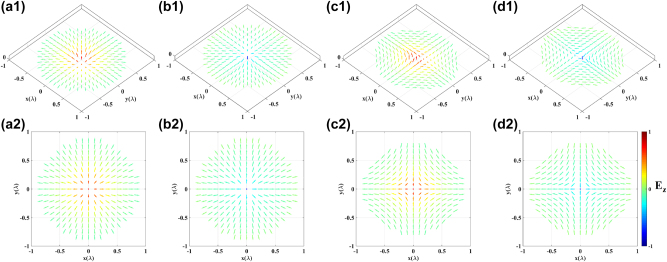
Meron and anti-meron topologies with different polarity. (a1, a2) and (b1, b2) are Néel-meron with negative polarity and positive polarity, respectively, (c1, c2) and (d1, d2) are Néel-antimeron with negative polarity and positive polarity, respectively.

In the previous instances, the oscillation polar angles of the dipoles at the origin and outermost ring are opposite, indicating that they undergo a complete flip from the inner to the outer region. However, if only a *π*/2 flip is experienced, it results in the formation of a meron-type topology of electric-field vectors. In this context, we set $\theta_{d\text{\_}1}^{\prime }=\pi /10$ , $\theta_{d\text{\_}2}^{\prime }=2\pi /10$ , $\theta_{d\text{\_}3}^{\prime }=3\pi /10$ , $\theta_{d\text{\_}4}^{\prime }=4\pi /10$ and $\theta_{d\text{\_}5}^{\prime }=\pi /2$ , with vorticity *v* = 1 or −1, and helicity *φ*_*r*_ = 0. Based on these parameters, we generated meron and anti-meron topologies formed by electric-field vectors, as shown in Figure 6(a1), (a2), (c1), and (c2). Moreover, if we change the polar angles to their complementary angles, the polarity will be inversed, meron and anti-meron topologies with positive polarity are illustrated in Figure 6(b1), (b2), (d1), and (d2). Their corresponding skyrmion numbers are −0.5055, 0.4995, 0.5033 and −0.4926, respectively. From Figures 3 [Fig j_nanoph-2023-0741_fig_004] [Fig j_nanoph-2023-0741_fig_005]–6, it is evident that by adjusting the oscillation directions of each dipole within the MCAD model, it is straightforward to generate diverse electric-field vector topological textures on the focal plane.

### 3.3 Optical skyrmions and metrons in prescribed plane

**Figure 7:**
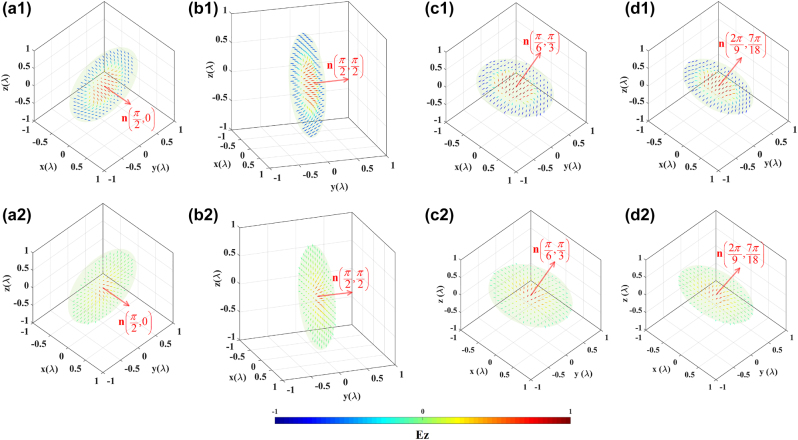
Optical skyrmions and merons formed by electric-field vectors in specified plane with $\left(\theta_{0},\varphi_{0}\right)$ equal to (a) $\left(\pi /2,0\right)$ , (b) $\left(\pi /2,\pi /2\right)$ , (c) $\left(\pi /6,\pi /3\right)$ and (d) $\left(2\pi /9,7\pi /18\right)$ .

In the third instance, we further demonstrate that our proposed model can generate optical skyrmions and merons located on arbitrary planes. This is achieved by adjusting the *x*′–*y*′ plane of the local Cartesian coordinate system, that is, the spatial orientation $\left(\theta_{0},\varphi_{0}\right)$ of **n**. Without loss of generality, using the Néel-type skyrmion and meron as references, we tailor the parameters $\left(\theta_{0},\varphi_{0}\right)$ as follows: $\left(\pi /2,0\right)$ , $\left(\pi /2,\pi /2\right)$ , $\left(\pi /6,\pi /3\right)$ and $\left(2\pi /9,7\pi /18\right)$ . By employing the same computational procedures, three-dimensional focal field data in the vicinity of the focal region is obtained, and the three-dimensional electric-field vector distribution on the specified plane (its normal direction are marked by the red elongated arrow) are plotted, as shown in Figure 7. The line-of-sight directions of Figure 7 are determined by their azimuth and elevation angles. For Figure 7(b1) and (b2), the azimuth angle of the line-of-sight is 7*π*/18, and the elevation angle is *π*/9. As for the other subfigures, the azimuth and elevation angles of the line-of-sight are both set to *π*/4. Figure 7(a1)–(d1) and (a2)–(d2) represent Néel-type skyrmions and merons located on different planes, respectively. Their corresponding skyrmion numbers are (−0.9190, −0.9189, −0.9049, −0.9065) and (−0.4912, −0.4885, −0.4713, −0.4673). The skyrmion numbers are slightly smaller than those in Figures 3 and 4, which were located at the focal plane, primarily due to the deviation introduced by extracting specified planes from three-dimensional space. Clearly, we have successfully generated Néel-type electric-field vector skyrmions and merons on the prescribed planes.

### 3.4 Comprehensive engineering of the focused optical topology

**Figure 8:**
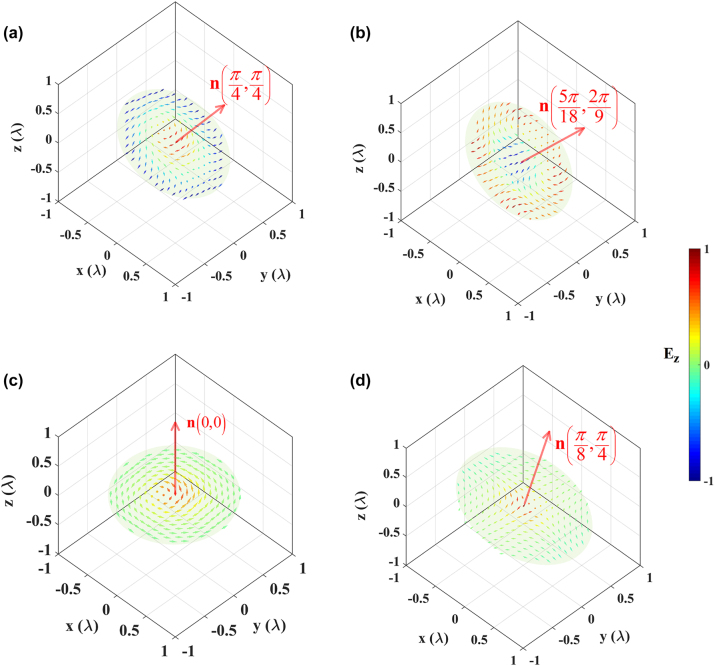
Comprehensive engineering of the focused optical topology. (a) Bloch-skyrmion with $\left(\pi /4,\pi /4\right)$ , *p* = −1 and *v* = 1; (b) anti-skyrmion with $\left(5\pi /18,2\pi /9\right)$ , *p* = 1 and *φ*_*r*_ = 0; (c) Bloch-meron with $\left(0,0\right)$ , *p* = −1 and *v* = 1; (d) anti-meron with $\left(\pi /8,\pi /4\right)$ , *p* = −1 and *φ*_*r*_ = 0. The azimuth and elevation angles of the line-of-sight are both set to *π*/4.

As a final demonstration, we design a comprehensive engineering endeavor devoted to the topologies of focused fields, encompassing the simultaneously tailor the topological texture of electric-field vectors on prescribed two-dimensional planes. Without loss of generality, we specified four sets of parameters, namely (skyrmion, *p* = −1, *v* = 1, *φ*_*r*_ = *π*/2, *θ*_0_ = *φ*_0_ = *π*/4) (anti-skyrmion, *p* = 1, *v* = −1, *φ*_*r*_ = 0, *θ*_0_ = 5*π*/18, *φ*_0_ = 2*π*/9) (meron, *p* = −1, *v* = 1, *φ*_*r*_ = *π*/2, *θ*_0_ = *φ*_0_ = 0) and (anti-meron, *p* = −1, *v* = −1, *φ*_*r*_ = 0, *θ*_0_ = *π*/8, *φ*_0_ = *π*/4), computed the data for the 3D focused fields. The 3D distribution of electric-field vectors on the specified planes (with their normals indicated by the red elongated arrows) are depicted in Figure 8. Figure 8(a)–(d) represent the comprehensive engineering of the Skyrmion and meron topologies, respectively. Their skyrmion numbers are denoted as −0.9035, −0.9120, −0.4756, and 0.4635. Through these instances, we illustrate that by adjusting the normal direction of the proposed MCAD array planes and the oscillation direction of each dipole, we can conveniently and effectively generate the corresponding electric-field vector topological textures on the desired planes.

## 4 Conclusions

In conclusion, we have demonstrated the full control of the topological type of optical skyrmions and merons formed by electric-field vector in highly confined light fields, where the topological planes can also be adjusted arbitrarily. Based on the presented model, we can effectively tailor the topological texture of the focused optical field and its normal orientation by adjusting the oscillation direction of each dipole in the MCAD and the direction parameters $\left(\theta_{0},\varphi_{0}\right)$ of the array. The proposed method significantly increases the degrees of freedom in controlling the electric-field vector based topologies of tightly focused optical field, offering potential applications in studying manipulation of optical topological properties at micro-nanoscale, as well as exploring novel phenomena that may arise from the interaction between skyrmions and materials.
